# Copper Amalgams

**Published:** 1889-01

**Authors:** 


ARTICLE V.

### COPPER AMALGAMS.

As it has been stated in the professional journals, on the authority of some prominent dentists, that copper amalgam does not shrink, and as this was quite contrary to my own experience, published by this society some years ago, I have undertaken a more extended series of experiments, the result of which is now before you.

I have tried four modes of ascertaining the shrinkage, viz., stopping, inserted in celluloid, human teeth, glass tubes, and the specific gravity test. The latter is the only one I consider of much value, and it was introduced by Mr. Charles Tomes many years ago. The reason why the other modes are objectionable is that the co-efficient of expansion is not the same as that of a human tooth, glass probably coming the nearest.

We have here twenty-four experiments with tubes, twenty-one of which are with copper amalgam, while the remaining three are with silver tin alloy. You will notice that all the copper amalgams have allowed the red ink to pass them, while the silver alloy has shown itself to be fairly tight.

—TABLE OF SHRINKAGE.—

*Specific Gravity List.*

| | | | |
|---|---|---|---|
| 5 | Specimens of A | — — — .65 | Copper |
| 1 | " " A | — — — .54 | |
| 2 | " " B | — — — .66 | |
| 4 | " " C | — — — .61 | |
| 1 | " " E | — — — .35 | Silver Alloys |
| 2 | " " F | — — — .15 | |
| 1 | Specimen of Silver Coin | — — — .21 | |
| 1 | " of Copper Amalgam and Silver Fillings | .48 | |

Masses tested weighed ten to twelve grammes, kept for six days at a temperature of one hundred degrees.

We have on the wall a table which I have drawn up of the absolute shrinkage of some nine or ten specimen of copper amalgam. When I brought before you similar tables some time ago, the accuracy of my experiments was doubted, because a very remarkable result was very fully shown. The earlier observations have, however, been quite corroborated by the more recent ones. You will notice that the amalgam does most of its shrinking within the first twenty-four hours, and that subsequently it seldom remains the same for any length of time, shrinking and expanding in a small but measurable quantity. To decrease the probable errors which might arise in this delicate form of investigation, I have used large masses of amalgam. In the earlier experiments I took less than two grammes, while in the present ones I used ten to twelve grammes. There is a fair amount of agreement in the shrinkage of different specimens of copper amalgam, 60 to 80 milligrams in 10,000.

TABLE SHOWING DAILY FLUCTUATION.

| | November 6 | November 7 | November 8 | November 9 | November 10 | Nov'ber 12 | Nov'ber 13 | Maximum Shrinkage. |
|---|---|---|---|---|---|---|---|---|
| No. 1 — | 12.041 | 12.113 | 12.117 | 12.116 | 12.114½ | 12.114½ | 12.115 | 76 |
| " 2 — | 12.010 | 12 081½ | 12.085 | 12.087 | 12.085 | 12.088 | 12.089 | 79 |
| " 3 — | 12.015 | 12.087½ | 12.095 | 12.092 | 12.098 | 12.098 | 12 101 | 86 |
| " 4 — | 12 010 | 12.080 | 12.085 | 12.087 | 12.093 | 12.094 | 12.098 | 88 |
| " 5 — | 12.016 | 12.089 | 12.087 | 12.089 | 12.091 | 12.093 | 12.095 | 79 |

The above represents in milligrams the daily change, increase in weight, showing shrinkage.

As a matter of comparison, I have combined with this table similar tests, with the same alloy used in the tube experiments. You will see that the percentage of shrinkage in copper amalgam is from 5 to 7 per cent. of the mass, while with one of the two specimens of silver alloy it is a trifle over 3 per cent., the other a little more than 1 per cent., or, in other words, the best copper amalgam shrinks four times as much as the silver alloys used.

Now we come to the question of edge strength. This table shows the breaking strain of the several amalgams already alluded to. The breaking strain was obtained by placing small amalgam bricks, one millimetre in thickness by four in breadth and twenty-five in length, in a crushing apparatus I made for the purpose. These bricks are formed in a steel mould, which we have upon the table. Five bricks of each kind of amalgam were tried, and each brick gave me from five to ten breakages. You will notice the enormous difference that exists between the maximum and minimum, some bricks giving way at a pressure of less than an ounce, while others gave me as high as seven pounds. The difference in strength of the amalgam bricks is owing more to the manner of treatment than to the difference in kind. To get the best results, the mercury should be fully driven out of the mass before it is broken up in the mortar. If the ordinary directions are followed and the spoon removed from the heat on the appearance of the globules, it will be found that the amalgam is comparatively weak. It is better to drive off an excess of mercury by overheating and subtracting, subsequently adding what may be necessary to obtain the proper plasticity, than it is to allow some of the combined mercury to remain in the mass. The bricks on the table which are so weak are those made by underheating the specimens. One of the several specimens, both in the specific gravity and breaking strain tests, has been heated and worked over six consecutive times, and I find little, if any, injury resulting.

BREAKING STRAIN.

*Bricks 1m. x 4m. x 25 m.*

| Kind | No. of Experiments. | Aver. Strain in ounces. | |
|---|---|---|---|
| A— | 52 — | 12 : 15-16— | Home made Copper Amalgam |
| A— | 36 — | 4 : 3-16 — | " " " underheated |
| B— | 28 — | 33¼ — | " " " 6th heating |
| C— | 37 — | 38 — | Copper Amal. from manufacturer |
| D— | 30 — | 34⅛ — | " " " " |
| G— | 26 — | 41 : 3-16 — | " " " " |
| G— | 23 — | 41 — | " " " " |
| G— | 20 — | 34⅜ — | " " " " |
| E— | 27 — | 55⅛ — | Silver Alloy from manufacturer |
| E— | 23 — | 69 : 13-16— | " " " " |
| E— | 24 — | 23 : 15-16— | " " " " |
| F— | 35 — | 63 : 3-16 — | " " " " |
| F— | 4 — | 60 — | " " " " |
| F— | 17 — | 58⅜ — | " " " " |

It will be observed that the silver alloys tested give a better result, not only as regards shrinkage but edge strength. It has been stated in the test books that silver is the expanding element of silver alloys. In this series of experiments I have only tried one specimen of silver, and that is standard or coin silver mixed with mercury. As you see, I fail to get any expansion but a considerable amount of contraction. Of course, a single experiment does not prove anything, but I hope to go into the subject more fully.

I have tried a curious experiment in mixing coin silver fillings with plastic copper amalgam. The gravity test has shown a diminution in the amount of contraction.

Amalgams of all kinds are very uncertain bodies; there is a molecular change going on, apparently indefinitely, and although it may not be sufficient to be a serious practical objection, yet it certainly must be one cause whv amalgam fillings are not always aatisfactory.

The directions given by manufacturers of copper amalgam are that it shall be washed in dilute sulphuric acid and subsequently in water. You will see by the litmus paper that, when these directions are closely followed, the acid is not fully eliminated; after washing several times with water I wash with soap and water.

One word in regard to the manufacture. Copper amalgam is, as you know, made from the sulphate of copper; this is dissolved in water precipitated by the action of iron or zinc. I have handed round for your inspection two specimens, one done by aid of zinc, the other of iron. While the zinc one has a fairly good colour, the iron has produced a marked effect upon the other, the oxide of iron showing itself by its red appearance, One ounce of the crystals ought to produce an ounce of amalgam, costing sevenpence. The rapidity of precipitation is vastly increased by the aid of heat. I have not found by these experiments that the precipitation by iron gives, in any respect, better results than that of zinc, while the colour is certainly inferior.

Finally, gentlemen, it seems to me that it has been fairly proved that copper amalgam shrinks much more than some silver alloys, and that it has also less strength, but, at the same time, it is undoubtedly a material of very considerable value as an antiseptic in the stopping of teeth of poor structure.

Mr. W. E. Harding (Shrewsbury) asked whether it made any difference whether the copper was precipitated with iron or zinc?

Mr. Amos Kirby (Bedford) said that the results which he had obtained from his own experiments were different from those of Dr. Elliott. In all cases he found silver amalgams expand considerably; that precipitated silver and coin silver both expanded—when placed in the tube they either split the tube open or projected from the orifice. When measured with a micrometer some showed so much expansion as 1 in 25 of the diameter; the only way he could account for the difference between Dr. Elliott's results and his own was that he (Dr. Elliott) spoke of his silver amalgam containing a small amount of copper; he (Mr. Kirby) found that the addition of any other metal modified the action considerably.

Dr. Elliott, in reply, said, with regard to Mr. Hardings' question, he had never used iron, and therefore could not say. With regard to the expansion of silver, he was aware that it was an accepted opinion that it *did* expand, but he could only say that he had made some 300 experiments simply hunting for expansion and had not found it. Of course, he could only speak from his own experience; others might have been more successful. Then, with regard to what Mr. Kirby had said, the fact that he (Dr. Elliott) used silver and copper, while Mr. Kirby used silver only, would be, he thought, a possible, but not a probable, explanation of the difference of their results.—*Odontological Society, London Dental Record.*

